# A Transcriptome Derived Female-Specific Marker from the Invasive Western Mosquitofish (*Gambusia affinis*)

**DOI:** 10.1371/journal.pone.0118214

**Published:** 2015-02-23

**Authors:** Dunja K. Lamatsch, Sofia Adolfsson, Alistair M. Senior, Guntram Christiansen, Maria Pichler, Yuichi Ozaki, Linnea Smeds, Manfred Schartl, Shinichi Nakagawa

**Affiliations:** 1 University of Innsbruck, Research Institute for Limnology, Mondsee, Austria; 2 Uppsala University, Department of Evolutionary Biology, Evolutionary Biology Centre, Uppsala, Sweden; 3 University of Otago, Department of Zoology, Dunedin, New Zealand; 4 University of Sydney, Charles Perkins Centre / School of Biological Sciences, Sydney, New South Wales, Australia; 5 University of Würzburg, Physiological Chemistry, Biocenter, Würzburg, Germany; Temasek Life Sciences Laboratory, SINGAPORE

## Abstract

Sex-specific markers are a prerequisite for understanding reproductive biology, genetic factors involved in sex differences, mechanisms of sex determination, and ultimately the evolution of sex chromosomes. The Western mosquitofish, *Gambusia affinis*, may be considered a model species for sex-chromosome evolution, as it displays female heterogamety (ZW/ZZ), and is also ecologically interesting as a worldwide invasive species. Here, *de novo* RNA-sequencing on the gonads of sexually mature *G*. *affinis* was used to identify contigs that were highly transcribed in females but not in males (i.e., transcripts with ovary-specific expression). Subsequently, 129 primer pairs spanning 79 contigs were tested by PCR to identify sex-specific transcripts. Of those primer pairs, one female-specific DNA marker was identified, Sanger sequenced and subsequently validated in 115 fish. Sequence analyses revealed a high similarity between the identified sex-specific marker and the 3´ UTR of the aminomethyl transferase (*amt*) gene of the closely related platyfish (*Xiphophorus maculatus*). This is the first time that RNA-seq has been used to successfully characterize a sex-specific marker in a fish species in the absence of a genome map. Additionally, the identified sex-specific marker represents one of only a handful of such markers in fishes.

## Introduction

Systems of sex determination attract considerable scientific attention, partially due to the great variety of mechanisms that operate among different species. In general, the identification of sex-specific or sex-biased genes can shed light on sex determination, as well as other biological phenomena such as sexual dimorphism and sex-specific selection. In vertebrates, various chromosomal sex determination systems have evolved. The most extensively studied systems are male heterogamety (XX/XY system) in mammals and female heterogamety (ZZ/ZW system) in birds. These vertebrates generally have highly differentiated sex chromosomes, where the X and Z chromosomes are large and gene rich, whereas the Y and W chromosomes (those specific to the heterogametic sex) are smaller, highly heterochromatic and, for the most part contain only a few functional genes. This heteromorphism is thought to be due to degeneration, as result of non-recombination between sex-chromosomes in the heterogametic sex [1,2]. In contrast to mammals and most birds, many other vertebrates, have no cytogenetically distinct sex chromosomes (for an overview see Ellegren [3]), a factor that makes them valuable in evolutionary/genetic studies as they may represent species with young sex chromosomes (i.e. where degeneration of the sex-chromosome specific to the heterogametic sex has not yet occurred) or systems with halted Y/W degeneration.

Fish species are a particularly attractive group in which to study sex chromosomes because such taxa appear to have independently evolved a variety of sex determination systems [4]. Sex determination systems vary between closely related fish taxa [5–9] often without clear phylogenetic patterns [10,11], and may even vary within the same population [12]. Although most teleost species studied do not display differentiated sex chromosomes [13],an extreme diversity of sex determination systems can be found. Gonochoristic and hermaphroditic species are relatively common, and sperm-dependent parthenogens are also known to exist. The factors that initiate differentiation of phenotypic sex also vary highly, ranging from behavioural or environmental factors to strict genetic ones. Where genetic factors do determine sex in teleost fishes, those factors can involve monogenic or polygenic systems [14,15], as well as a variety of sex chromosome systems; e.g. single (XX/X0, XX/XY, ZZ/WZ) and multiple sex chromosomes (X_1_X_1_X_2_X_2_/X_1_X_2_Y, XX/XY_1_Y_2_, ZZ/ZW_1_W_2_) [13,16–19].

The Western mosquitofish, (*Gambusia affinis*; Baird and Girard 1853), originates from North America but was distributed throughout the world for the biological control of mosquitos. However, the species is now largely regarded as pest in introduced locations [20–24]. *G*. *affinis* displays female heterogamety (ZW/ZZ), and is one of the few species where the W chromosome is the largest chromosome of the karyotype and hence, much larger than Z [25–27]. Its closely related sister taxa, *G*. *holbrooki*, is almost indistinguishable from *G*. *affinis* on the basis of morphology alone, but has homomorphic sex chromosomes with a contrasting XX/XY sex determination system [25]; i.e. male heterogamety.

Poeciliids, and the *Gambusia* species described above in particular, make excellent model systems in which to study the evolution of sex-determining systems, and sex chromosomes specifically. A key step in the study of sex-determination systems is the early identification of an individual’s phenotypic sex. However, diagnosis of phenotypic sex in live early-stage embryos or fry on the basis of morphology is often not possible in (Poeciliid) fishes. Typically, males only develop secondary sexual characters such as the gonopodium (a highly specialized insemination apparatus modified from the anal fin) at onset of testosterone production after puberty [28]. Size is also not a reliable character with which to differentiate the sexes, due to individual variation in growth-rate and development. Thus, a sex-specific marker is required to identify sex in juveniles at early life-history stages (i.e. prior to morphological separation of the sexes). In addition to early identification of sex, markers that unequivocally indicate the genotypic sex of an individual (i.e. WZ vs ZZ) allow for the detection of naturally sex-reversed individuals, and the subsequent study of the causes of such aberrant sexual development.

The identification of sex-specific markers in fish has, however, proved problematic. Recombination between sex chromosomes is common in organisms that either lack heterogamety, or have sex chromosomes with limited differentiation (see [29–31]). Absence of recombination between heterogametic sex chromosomes leads to accumulation of repetitive DNA on the sex chromosome specific to the heterogametic sex (i.e. W or Y). This accumulation makes it difficult to find the few genes solely located on the W (or Y), even with the use of modern techniques (i.e. next generation sequencing). New approaches are therefore necessary to identify sex chromosome specific sequences (see Chen et al. [32]). Here, we performed a non-targeted expression analysis using RNA-seq to identify female-biased loci in *G*. *affinis* potentially located on the W sex chromosome. This method was successful in identifying a female-specific molecular marker. This marker represents one of only a handful of such tools in non-model fish species.

## Materials and Methods

### Fish samples

Indigenous *G*. *affinis* (N = 44) from Mexico (Pena Blanca, Santa Cruz River system, north of Nogales, Sonora, Mexico; 25 females, 19 males) as well as introduced *G*. *affinis* (N = 71) from New Zealand, North Island (Chapel lake, Waikato University, Hamilton; 29 females, 42 males) were used for primer testing. Primers were also tested on G. affinis’ sister species, *G*. *holbrooki*, from Leninskoe (North-East of Bishkek, Kyrgyzstan; 21 females, 7 males). *G*. *holbrooki* is also a common model organism and hence, the applicability of our marker to that species would likely be of wide interest.

Field studies (i.e. collections) did not involve endangered or protected species. All fish were caught as juveniles by hand netting, and transported back to laboratories in their respective countries (Dunedin, New Zealand, and Würzburg, Germany). Fish were then raised to maturity in temperature-controlled rooms, at an average of 25°C and under a 12:12 light:dark cycle. No specific collection permissions were required for Kyrgyzstan or New Zealand as *G*. *affinis* and *holbrooki* are introduced, invasive fish species. The *G*. *affinis* strain from Mexico is a long-established aquarium strain that was collected prior to the existence of regulations for fishing (i.e. decades ago). That strain was first kept for fish hobbyists, and only recently transitioned in to scientific use.

This study was carried out in strict accordance with the recommendations in the ‘Guide for the Care and Use of Laboratory Animals’ of the National Institutes of Health. All protocols were approved by the Animal Ethics Committee of the University of Otago (Permit Number: 87/08) and the Animal Protection Officer of the University of Würzburg from the Veterinary Office of the District Government of Lower Franconia, Germany. The number of fish killed or fin-clipped is reported yearly for each species (fin biopsy according to authorization 55.2–2531.01–49/08). Animals were terminated by cervical dislocation, and all efforts were made to minimize suffering.

### lllumina HiSeq sequencing

Following the onset of maturity (i.e. sexual differentiation had occurred) 12 male and 12 female fish (*G*. *affinis*) from New Zealand were dissected and their gonads removed (testis from males and ovaries from females). Gonadal samples were stored in RNAlater (Ambion, Austin, Texas) following manufacturer’s instructions to prevent RNA degradation, and transported to Uppsala University for RNA extraction. Total RNA was extracted from gonads using the RNeasy Mini Kit (Qiagen, Sollentuna, Sweden) following the supplier’s recommendations. Before sequencing we pooled 12 male *G*. *affinis* into 6 groups each with two individuals, generating six ‘male-expression’ replicates. The same process was applied to 12 female *G*. *affinis*. Barcoded pools were then sequenced in two lanes of an Illumina HiSeq2000.

Sequencing libraries were prepared from 1–4 µg of total RNA according to the TruSeq RNA sample preparation guide #15008136 revA using reagents from the TruSeq RNA sample prep kit set A and set B v1 (Illumina, San Diego, CA). Briefly, poly-A containing mRNA was purified from 1.5 µg of total RNA using poly-T oligo attached magnetic beads, followed by fragmentation of the mRNA. First strand cDNA was synthesized using SuperScript III reverse transcriptase (Invitrogen, Carlsbad, CA) and random hexamers, followed by second strand synthesis according to the manufacturer’s reagents and protocols. The overhangs on the DNA fragments were end-repaired followed by purification using AMPure XP beads (Beckman Coulter, Brea, CA). An A-base was added to the blunt ends of the DNA fragments and adapters, and index tags for sequencing were ligated, followed by a new round of purification using AMPure XP beads. Libraries were amplified for 12–15 PCR cycles, followed by purification using AMPure XP beads. Library qualities were evaluated using the Agilent Technologies 2100 Bioanalyzer and a DNA 1000-kit. Adapter-ligated fragments were quantified by qPCR using the Library quantification kit for Illumina (KAPA Biosystems, Cambridge, MA) on a StepOnePlus instrument (Applied Biosystems/Life technologies, Carlsbad, CA) prior to cluster generation and sequencing. A 6–10 pM solution of the pooled libraries (see below) was subjected to cluster generation on a cBot instrument (Illumina Inc.). Paired-end sequencing was performed for 100 cycles in one lane using a HiSeq2000 instrument (Illumina Inc), according to the manufacturer’s protocols.

Base calling was performed on the instrument by RTA 1.10.36 and the resulting. bcl files were converted to Illumina qseq format with tools provided by OLB-1.9.0 (Illumina Inc.). To separate samples and PhiX control DNA sequenced in the same lane as the sample libraries, the qseq-files were de-multiplexed, allowing for one mismatch. Both de-multiplexing and mapping were done with CASAVA 1.7.0 (Illumina Inc.). Additional statistics on sequence quality were compiled from the base call files with an in-house script. Note that original raw reads have been deposited to NIH Short Read Archive, accession number SRP033398.

### *De novo* assembly and differential expression analysis

Raw sequencing reads were filtered for unique pairs and trimmed, removing bases with quality scores <25, using ConDeTri v1.0 [33]. We then checked that there were no signs of contamination or sequence biases with FastQC v0.7.2 (http://www.bioinformatics.babraham.ac.uk/projects/fastqc/). Reads were assembled *de novo* with Oases v0.1.21 [34] defining a *k*-mer size of 33. An evaluation of *k*-mer 17–61 showed that this *k*-mer size optimized the relationship between contig N50, number of medaka (*Oryzias latipes*) genes (Ensembl 63) to which contigs align using reciprocal BLAST (in house script) and *G*. *affinis* contig coverage of medaka genes. The coverage was calculated as (medaka gene length + average UTR length) /contig length excluding N’s. We used medaka genes to evaluate the *de novo* assembly, as this is the least divergent fully sequenced genome. Each pool was assembled separately. Contigs from all pools were then merged with Newbler v2.5.3 [35], which is designed to assemble longer reads.

We then mapped reads from each pool onto the contigs using BWA version 0.5.9 [36], not allowing for multiple hits and defining a maximum insert size of 250bp. Differential expression analysis was conducted with *baySeq* v1.6.0 (*R* package version 1.2.0; [37]) where we normalized over library size and gene length. This Transcriptome Shotgun Assembly project has been deposited at DDBJ/EMBL/GenBank under the accession GBAE00000000. The version described in this paper is the first version, GBAE01000000.

### Female specific expression

Putative female specific contigs were identified based on expression profiles in males and females. We chose contigs for downstream analysis that were constructed exclusively from reads derived from female samples, that were >500bp and with a likelihood of differential expression of 1 (calculated in BaySeq).

### Primer design

We then designed primers to test the female-only-expressed sequences identified as candidates for sex specific markers. Primers were designed for 79 contigs, excluding 7 contigs, which were confirmed to be subject to bacterial contamination.

All primers were designed with Primer3Plus [38] using default settings, except the following: primer T_m_: min. 59, opt. 60, max. 61; max. T_m_ difference: 1. Advanced settings: Max Poly-x: 3; GC clamp: 1; product size: min. 480, opt. 500, max. 520. Restricting product size to 500bp seemed like a feasible approach that would cover introns that might enlarge the product manyfold.

In a further approach, seven additional primers were designed for three *G*. *affinis* transcriptome contigs identified by BLAST to be sex-linked EST markers from *Oryzias hubbsi* clone br8179 (Genbank accession number AU171840), OLb06.11h (AB095500), and OLb22.11h (AV670414) [7]. *O*. *hubbsi* has, in similarity to *G*. *affinis*, a ZZ/ZW sex-chromosome system with a morphologically larger W than Z [7]. Each primer was tested simultaneously on three females and two males. A positive control was chosen from the transcriptome on the basis to be highly expessed in both sexes (contig15716X; S2 Table). This sequence refers to cathepsin K in the 5´UTR region and exon1 of *X*. *maculatus* (ENSEMBL). To avoid overlapping product sizes in the multiplex PCR, the primers for the positive control (15716_F:GGGGAACAAGGGTTACGTCT, 15716_R:ACCACAGGAAGGGAGGAACT) were designed to result in a smaller product than all other products (i.e. 259bp).

All candidate sexing primers were tested by PCR amplification on genomic DNA. Primer pairs were scored based on their ability to produce bands from all female templates that differed from the bands produced from all male templates. Primer pairs with identical results on male and female templates were scored as non-specific. If a given primer pair amplified a different pattern in males and females it was considered sex-specific. Primers showing the slightest difference between male and female were tested again on 10 fishes from Mexico and New Zealand, respectively (5 females, 5 males) without positive control.

### DNA extraction

DNA was extracted from fish organs (brain, liver, gills, kidney) or muscular tissue by DNeasy Blood&Tissue Kit (Qiagen, Vienna, Austria) and diluted to 50ng/μl prior to PCR amplification.

### PCR conditions

For primer testing, a multiplex PCR Kit (Qiagen) was used following Kenta et al. [39] with minor adjustments. PCR was carried out in 10 µl on a Mastercycler (Eppendorf, Vienna, Austria) with two primer pairs each. The PCR thermocycling conditions were identical for all multiplex sets: an initial denaturation step at 95°C for 15 min to activate the hot start Taq polymerase, followed by 10 touchdown cycles of denaturation at 94°C for 30 s, annealing at 60–51°C (decreasing by 1°C per cycle) for 90 s, and extension at 72°C for 90 s, followed by 40 subsequent similar cycles with annealing at 50°C for 90 s, finally followed by an extension at 60°C for 10 min. The PCR products were separated on 1.5% agarose gels, 0.5% TBE at 5V/cm, ethidium bromide stained and photographed under UV light. Amplification patterns were analysed by eye. For female specific PCR products the same conditions were used but without touchdown cycles (T_a_ = 55°C) and with reduced number of cyles (i.e. 30) and a normal Taq (Dream Taq, Thermo Scientific, Vienna, Austria).

### Cloning, sequencing, and sequence analysis

Female-specific bands were cut from the gel, cleaned with the QIAquick Gelextraction Kit (QIAGEN) and sent for sequencing according to the sample submission guide for value read tubes (Eurofins MWG Operon, Ebersberg, Germany). Several bands from male PCR products were cloned into pGEM-T Easy Vector Systems (Promega, Mannheim, Germany), and transformed into competent cells of *E*. *coli* DH5α strain (Invitrogen Life Technologies, Vienna, Austria) according to the manufacturer´s instructions and sent for sequencing.

The putative aminomethyl transferase gene of *G*. *affinis* was amplified by different primers designed from the sequence information of *X*. *maculatus amt*-gene (ENSXMAT00000019396) and sent for sequencing (see primers in Table 1).

**Table 1:** PCR primers spanning the aminomethyl transferase (***amt***) gene of ***X*. *maculatus*** for sequencing in ***G*. *affinis***.

Oligo name	Product length [bp]	Primer _F	Primer_R	males	females
Exon1	126	ATGTGGGCTCGGGTTACG	GGCACTGAAATCCCGTCTCT	+	+
Exon2	151	ACCACTCTCTTTGACTTCCACAG	GGAGCATGTGGCTGACATC	+	+
Exon2_Intron3	156	GACCAAAATCCACGGAAAAG	GCTACAGATCATCTTGTTGGAATC	+	+
Exon2_3	358	ACTTCCACAGGAACAATGGTG	TCTGCGATATCTGCAACCAC	+	+
Intron3	1,386	GCTACAGATCATCTTGTTGGAATC	TTCATAAGAGCCGAGTCTTTGTC	+	+
Intron3_Exon4	200	CTCCCCACTCCTTTTCCTTC	CGATTCCAGGCCAGTCAG	+	+
Exon4_5	266	AGACCGACCAGGGTTACCTC	CTCCAGGTCCACATCAAACC	+	+
Exon6	104	CATGGCTCAGGTGCTTCAG	TCACCCTGCAGTCAGGAATAC	+	+
Exon7	172	TCTGTTCCTCGTTCCAGAGTG	TCCAAACAAGACTGGCTTCC	+	+
Exon8	100	ATTGTGCCGCAAATCAAAG	CATCAGGGCTCAGGATGG	+	+
Exon9	105	AACGTCGCCATGGGTTAC	GGGCATCTTGCTGACAATG	+	+
Exon9_UTR	935	AACGTCGCCATGGGTTAC	CCCTCATATTTCAACCAATGTG	**-**	**+**
Gaf88	501	GGGACACTAGAGCCCACAAA	CAACCAATGTGGAGCATTTTC	**-**	**+**
**Total**	**6,498**				

Primers spanning from exon 9 into the 3´UTR and Gaf88 only amplified in females. T_m_ 60.0±1.0°C.

Sequence editing was performed using the computer program CodonCodeAligner 4.0 (Centerville, MA, USA). Sequences were subjected to BLASTN [40] searches at the National Center for Biotechnology Information (NCBI), using nucleotide collection (nr/nt) or BLAT [41] searches in ENSEMBL against the platyfish genome (*Xiphophorus maculatus*).

### Species confirmation

To genetically confirm our specimens were indeed *G*. *affinis* and not the closely related *G*. *holbrooki*, we designed species-specific cytochrome oxidase subunit 1 (COI) primers: We downloaded the COI sequences (652bp) of 6 *G*. *affinis* and 10 *G*. *holbrooki* from Genbank, aligned them using Multalin (v 5.4.1; [42]) and identified the base positions where the sequences differed between the species. Primers were designed to cover regions with 2 and 3 nucleotide differences, respectively, between the two species with the 3´end of the primer ending on one of the nucleotide differences (in bold): COI_GafF: TAATTGGTGCCCC**C**GACAT**G**; COI_GafR: GGAG**G**ACAGCTGTAATTAGGACTGC**T**CA**C** (S1a Fig). With a T_m_ of 66ºC and 68ºC, respectively, the primers amplify a 327bp product at 66°C annealing temperature in *G*. *affinis* but not in *G*. *holbrooki* (T_m_ 60.4 / 67.4 ºC) (S1b Fig).

The following amplification protocol was used: 50ng DNA, HotstarTaq (Qiagen) with 1.5mM MgCl_2_ and 5 µM of each primer. The PCR amplification was performed in a total volume of 10 µl for 15 min at 95°C, followed by 32 cycles of 30 s at 94°C for, 30 s at 66°C, and 45 s at 72°C, with a final elongation step of 10 min at 60°C. The PCR products were separated on 1.5% agarose gels/0.5x TBE at 5V/cm ethidium bromide stained and photographed under UV light.

To verify our species determination approach, we amplified the COI fragments at lower temperatures (S1b Fig) and sequenced products from both species. The resultant sequences were 100% concordant with the voucher sequences (S1 Table).

### Species divergence

The species divergence time of *G*. *affinis* and *G*. *holbrooki* was estimated from mitochondrial DNA sequence difference values at the control region (acc. numbers: AY224097, GU188431) and cytochrome b gene (acc. numbers: EF017514, GU183104), respectively. Sequence difference values were 6 out of 396bp (1.52%) for control region, and 41 out of 876bp (4.68%) for cytochrome b (NCBI BLAST alignment, megablast; [41]).

To estimate the minimal and maximal divergence times of the two species, the sequence difference values were divided by the fastest and slowest rates of known calibrated molecular clocks for mitochondrial DNA in teleosts (i.e. 0.0076–0.0036 changes/site/Myr for cytochrome b, and 0.044–0.004 changes/site/Myr for control region) [43].

## Results

### NGS data analysis

Per pool, the number of unique reads with quality >25 ranged from 14,658,731 to 47,081,412 (average 31,459,508). The number of contigs constructed ranged from 43,467 to 82,803 (average 64,734), total contig length from 27,788,480 bp to 63,525,980 bp (average 47,305,990 bp) and N50 from 961 bp to 1,515 bp (average 1,196 bp). Merging contigs with Newbler then resulted in 47,347 contigs with a total contig length of 63,648,638 bp and a N50 of 2,496 bp. These contigs were then analysed for differential expression.

### Female specific expression

108 putative female-specific contigs were identified based on expression profiles in males and females. We excluded contigs which obviously showed contamination by bacteria according to Genbank (N = 7), and tested the remaining contigs (N = 79) until positive result. The supplementary S2 Table shows all contigs including positive control (contig15716X) and the three sex-linked EST markers from *Oryzias hubbsi*.

### Search for sex-specific sequences

**Fig 1:**
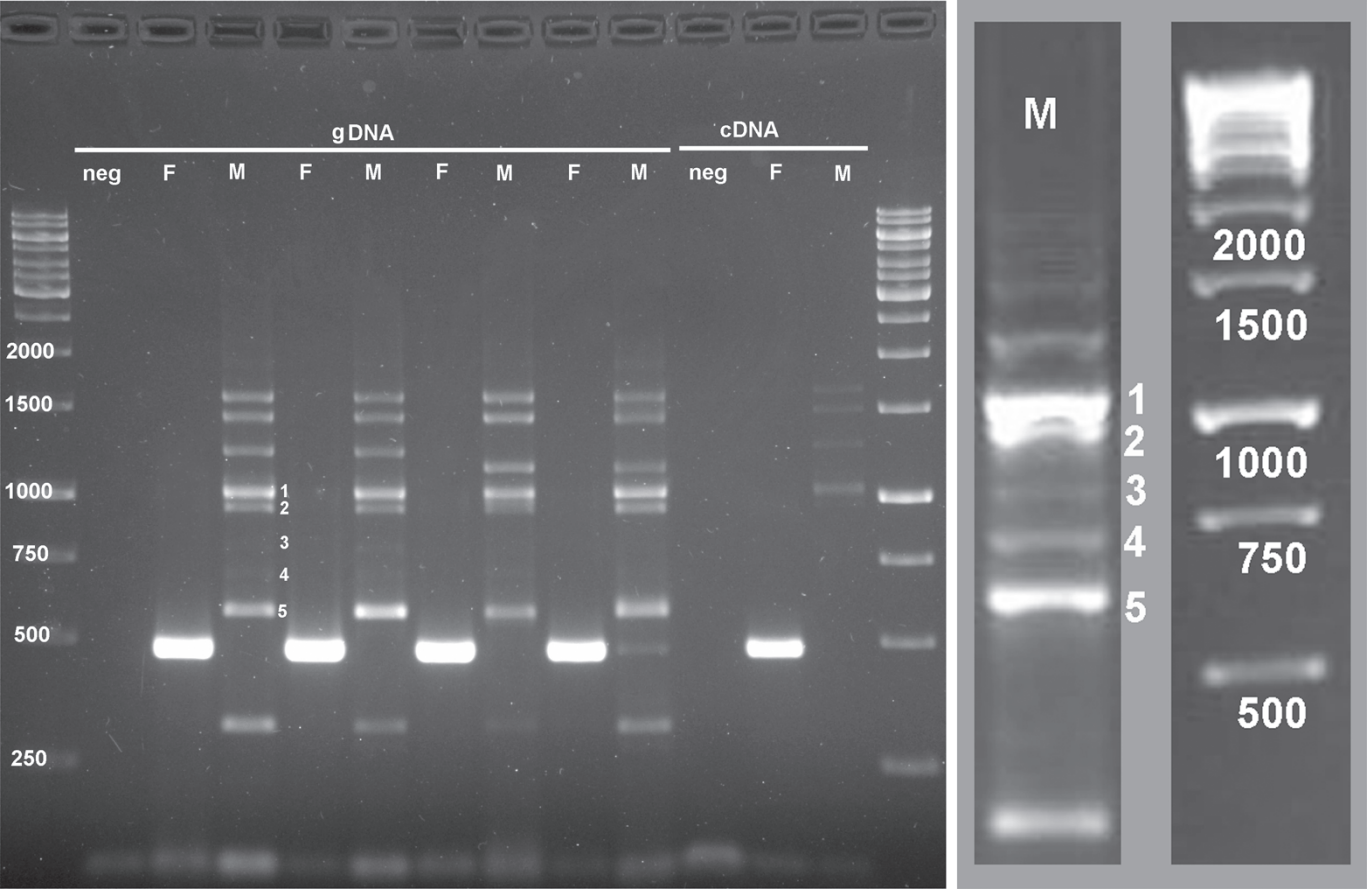
Sex-specific amplification of Gaf88. Sex-specific PCR amplification with primers specific to sequence contig23199X (Gaf88) from the transcriptome of *G*. *affinis*. Females (F) show a specific 500bp band identical to the original contig in genomic DNA (gDNA) as well as in cDNA, whereas males (M) do not show this band but a multiband-profile ranging from approx. 560–2000 bp. Male bands numbered 1–5 have been isolated and sequenced (enlargement). 1.5% agarose gel, 0.5%TBE, 5V/cm.

We tested 129 primer pairs from 79 contigs, covering a total of 61,763 bp, as well as 7 primer pairs derived from 3 sex-linked EST markers in *O*. *hubbsi* [7], which covered 3,202 bp. From a total of 136 tested primer pairs covering a total of 64,966 bp, we found one that differentially amplified male and female genomic DNA of *G*. *affinis*: Females showed a strong 500bp band, whereas males showed a multi-band profile (Fig. 1). The identified female-specific marker was termed Gaf88 and corresponds to contig23199X. This primer pair was tested on a total of 115 fishes: 25 females and 19 males from Mexico, and 29 females and 42 males from New Zealand. All but one of the tested individuals showed the banding pattern predicted by their phenotypic sex. When amplified with the same primers, the males and females of the sister species, *G*. *holbrooki*, gave a multi-band profile identical to that produced by male *G*. *affinis* (N = 7 and 21, males and females respectively; data not shown).

Based on transcriptome reads (see S2 Fig), the sequence of Gaf88 was revealed to be a 779 bp contig. Sequencing of the female amplified products (Fig. 1) showed a 100% match with the original contig sequence (501bp, N = 2). No significant hits were found in BLASTN (NCBI), but a BLAT search against the platyfish genome (*Xiphophorus maculatus*) in ENSEMBL revealed on average 93.1% similarity with a predicted aminomethyl transferase gene (*amt*, ENSXMAT00000019396; scaffold JH556705.1: 1,171,505–1,178,951) (of 771 from 779 bp) (S3 Fig, S3 Table). The sequence match is in the 3´ UTR of Xma *amt*.

The male sequences were mostly larger (approx. 560–2000bp) (Fig. 1). 31 cloned PCR products from two males were sequenced but gave no significant hits with either BLASTN (NCBI) or BLAT (ENSEMBL) (Genbank accession numbers KP179419-KP179449).

Primers designed to span the nine exons of *amt from X*. *maculatus* (ENSXMAT00000019396) amplified products in both, males and females, with no significant length differences. As expected, primers spanning from Exon 9 to the 3´UTR of *amt* (Exon9_UTR) as well as Gaf88 primers resulted in a product from females only (see Table 1).

Sequencing of all exons and introns from two males and two females resulted in a 6,498 bp consensus sequence (Genbank accession number KP113677), which showed 90% identity with *amt* from platy (93% query cover, E-value: 0) (S3 Fig, Table 1).

## Discussion

The identification of sex-specific markers can be a key step in understanding reproductive biology, genetic factors involved in sexual dimorphisms, mechanisms of sex determination and the evolution of sex chromosomes within and between species. Here, we generated the female-specific marker Gaf88 for the Western mosquitofish, *Gambusia affinis*, by screening sex-differentially expressed sequences from a transcriptome composed of pooled gonads.

To our knowledge, this is the first time that transcriptomes were successfully used to identify a sex-specific marker in a fish species. Although Hale et al. [44] attempted to discern a sex specific marker in sturgeon (*Acipenser fulvescens*) by massive parallel pyrosequencing of gonad transcriptomes, they ultimately failed to identify a sex-specific product from 73 candidate contigs. It seems that no method has yet been successful in identifying sex specific markers in sturgeon [45]. Given the falling price of transcriptomics many references can be found which describe the analysis of the transcriptomes of fish and list putative sex-related genes, but without diagnostic marker identification (in fishes e.g. Liu et al. [46] and Tao et al. [47] in tilapia; Shen et al. [48] in Asian arowana; Vidotto et al. [49] in Adriatic sturgeon; Sun et al. [50] in catfish).

As well as the approach that we describe here, a string of other methods have also been successfully used to identify sex-specific markers in fishes. Those methods include, subtractive cloning (e.g. Nakayama et al. [51], in *Leporinus elongatus*), randomly amplified polymorphic DNA (RAPD; e.g. da Silva et al. [52], in *Brycon amazonicus*; Xia et al. [53], in *Paramisgurnus dabryanus*; Vale et al. [54], in turbot), representational difference analysis (RDA; e.g. Sato et al. [55], in *Oryzias*), amplified fragment length polymorphism (AFLP; e.g. Olmstead et al. [56], in the fathead minnow, *Pimephales promelas*; Cui et al. [57] in *Takifugu rubripes*; Chen et al. [58], in the tongue sole, *Cynoglossus semilaevis*; Brunelli and Thorgaard [59], in the Pacific salmon), Restriction-site Associated DNA (RAD) sequencing (e.g. Palaiokostas et al. [60], in the Atlantic halibut *Hippoglossus hippoglossus)*, and genetic linkage map (Rondeau et al. [61], in sablefish *Anoplopoma fimbria*).

The female-specific marker we describe here identified sex in individuals from independent non-mixing populations (i.e. fish from Mexico and New Zealand). Among the 115 individuals subject to molecular sexing, we identified only one female that produced a negative amplification pattern following PCR with Gaf88. This fish was possibly a naturally feminized ZZ neo-female. Unfortunately, this individual was not available for cytogenetic analyses, as the presence or absence of W can easily be recognized in chromosomal metaphase spreads. In the future, our marker may be more widely applied to identify other such exceptional fish. Previous studies have suggested sex-determination to be relatively plastic in most teleosts, including *G*. *affinis* [62] (reviewed in Senior and Nakagawa [63] and Senior et al. [64]), thus naturally feminized or masculinized animals maybe widespread. In instances of sex-reversal identified by sex-specific marker, the karyotype may also be used to clarify the alternative hypothesis; namely that the sex-reversed fish was a recombinant and that the negative PCR result was the consequence of a W/Z sex chromosomal cross-over [65].

The sequence of Gaf88 shows a high similarity with the 3´UTR sequence of an ORF coding for an enzyme with homology to an aminomethyl transferase (*amt*) from a fish from the same family (Poeciliidae, *Xiphophorus maculatus*). This enzyme is a tetrameric protein of the “glycine cleavage” system. Glycine is not an essential amino acid but a neurotransmitter, and the breakdown of excess glycine is necessary for the normal development and function of nerve cells in the brain and spinal cord [66]. Due to its crucial biochemical role, it is not clear why the (likely) *amt-*gene should be differentially expressed in male and female gonads of *G*. *affinis*. The gene is present in males and females, as we have proven by sequencing, revealing a 90% identity with *amt* from *X*.*maculatus*. Based on these facts, two explanations for the lack of amplification of a product from male genomic DNA are identifiable to us: 1) differences in the primer binding sequence between W and Z or 2) a very large insertion in 3’ UTR of the Z-copy, which yields a product size that cannot be amplified by conventional PCR.

**Fig 2:**
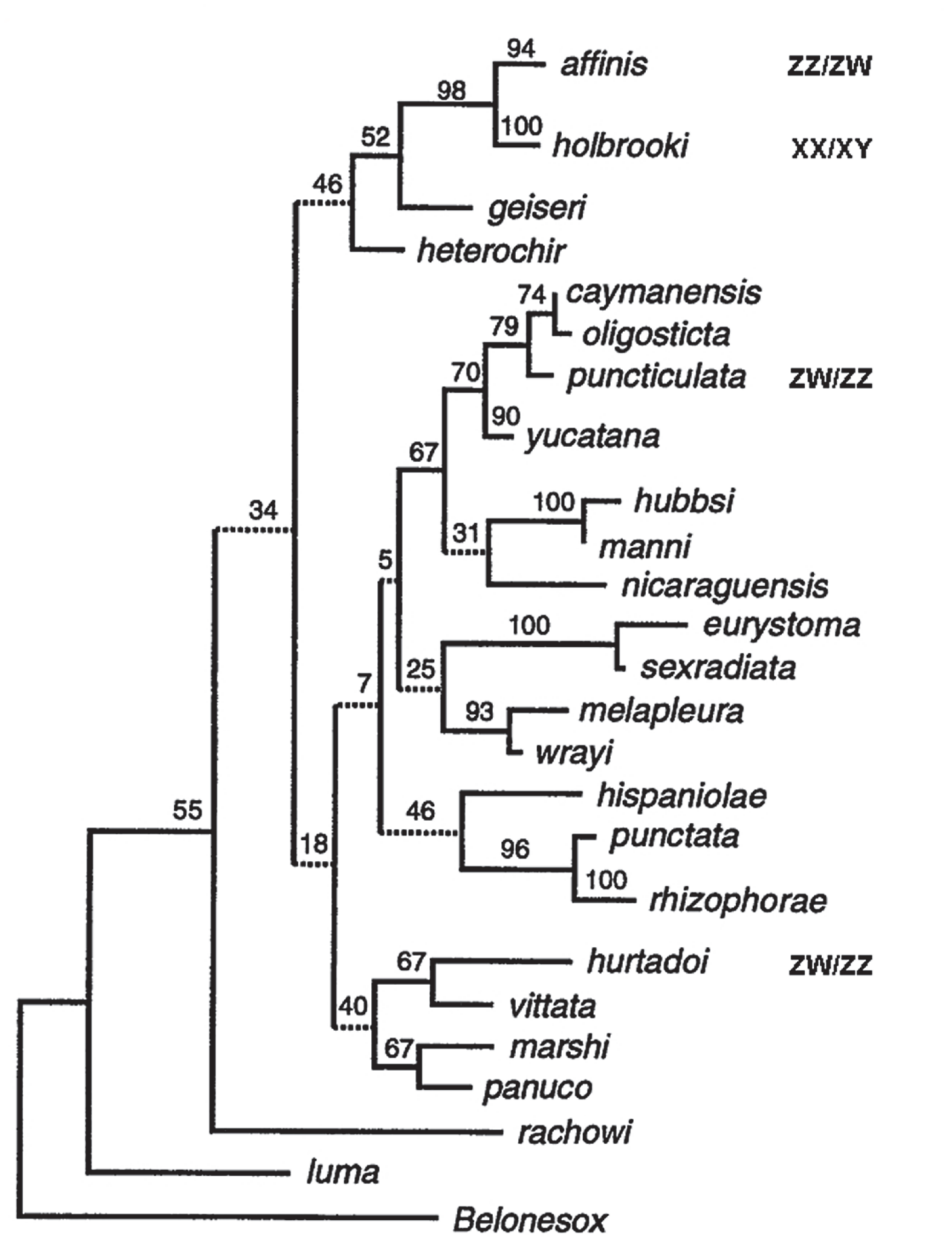
Phylogeny of *Gambusia*. A cladogram of the single most-parsimonious tree for *Gambusia* derived from up to 407bp of a segment of the mitochondrial cytochrome b gene. Where known, the sex determination mechanism is given. Oxford University Press grant permission for the requested material to be reused: Fig. 1 from Lydeard et al. [69].

According to Devlin and Nagahama [13] sex determination has been elucidated in only a few species of the genus *Gambusia*: an XX/XY system has been identified in *G*. *holbrooki*, whereas ZZ/ZW was found for *G*. *gaigei*, *G*. *puncticulata*, *G*. *hurtadoi*, *G*. *nobilis*, and of course *G*. *affinis* (Fig. 2; [26,67,68]). Since the sex determination system is not known for *G*. *heterochir* and *G*. *geiseri*, the sister clade to *G*. *affinis/G*. *holbrooki*, it is difficult to speculate about origin and evolution of the W chromosome in *Gambusia*. Testing Gaf88 widely within the genus may produce interesting insights in to the evolution of sex chromosomes in this group. Unfortunately, perhaps the most interesting species to which Gaff88 might be applied (i.e. *G*. *heterochir* and *G*. *geiseri*) were not available to us as these species are currently of a conservation concern. Here, we were only able test our marker in the sister species of *G*. *affinis*, *G*. *holbrooki* (XX/XY). Although, we note that *G*. *holbrooki* is another common model organism, thus the outcome of the applicability of our marker to that species will likely be of some interest. Both, male and female *G*. *holbrooki* gave a banding pattern identical to that produced by male *G*. *affinis*, indicating that the female specific sequence is absent from *G*. *holbrooki*. It cannot be concluded whether: 1) the marker is specific to a newly derived W chromosome after the separation of the two sister species [69,70] or 2) whether there was an ancestral ZW/ZZ system in the group [(*affinis*, *holbrooki*) (*geiseri*, *heterochir*)], and *G*. *holbrooki* might have lost the W, developing a new XY system. A phylogenetic analysis in anurans suggests, however, that shifts from ZW to XY are more frequent than the reciprocal process (for a review see Bachtrog et al. [71]).

We estimated the divergence time of *G*. *affinis* and *G*. *holbrooki* using mitochondrial DNA sequences based on the fastest and slowest rates of known calibrated molecular clocks for mitochondrial DNA in teleosts [43]. The differences between cytochrome b sequences (0.0076–0.0036 changes/site/Myr) give a minimal age for the W-chromosome of *G*. *affinis* between 6.16 and 13 million years. The calculation for the control region (0.044–0.004 changes/site/Myr) gives a minimal age between 0.35 and 3.8 million years, always assuming the sex chromosome turnover between XX/XY and ZW/ZZ has evolved in parallel with the species divergence.

In contrast to most species where the sex-limited chromosome (W or Y) is smaller than the respective Z or X chromosome, the W-chromosome is the largest of the karyotype in *G*. *affinis*. This might indicate that genetic degeneration has hardly occurred; an assumption that is supported by an indifferent chromosome staining with DAPI or mithramycin (AT and CG-specific stain, respectively, for detection of highly repetitive DNA blocks; Schartl, Nanda, Schmid pers. comm.). A comparative genome hybridization (male and female DNA on female chromosomes) might indicate that the p-arm of the W is still recombining with the Z chromosome due to a balanced hybridization pattern. However, the q-arm of W shows an overrepresentation of female DNA sequences excluding recombination between W and Z (Lamatsch et al., in prep.). It is thus crucial to identify the chromosomal location of the female-specific marker in *G*. *affinis*.

Until only recently, the complete sequence of a W chromosome in any system of female heterogamety remained elusive; mostly because a large portion of the initial chicken W chromosome assembly was later discovered to be misassigned [32]. Comparison of the relatively young tongue sole sex chromosomes with those of birds and mammals, however, now provides important insights into ZW sex chromosome evolution [72,73]. Such sequence data will be integral to a better understanding the evolution of non-recombining sex chromosomes that are not subject to the potent forces of sexual selection (i.e. female specific chromosomes; [3]).

Therefore, in the future we plan to perform chromosome sorting and whole chromosome sequencing of the W chromosome of *Gambusia affinis—*a unique model species where (1) the sex chromosome has evolved as the largest chromosome of the karyotype [26], and (2) the closest relative has homomorphic chromosomes with an XX/XY sex determining system [25].

There remains a lack of knowledge concerning the roots of genetic sex determination, especially in lower vertebrates. As we have shown here, RNA-seq on transcriptomes may be a valuable tool to locate and isolate genetic markers for sex-specific regions of the genome.

## Supporting Information

S1 Table NCBI BLAST of amplified COI sequences for species confirmation. (DOCX)

S2 Table Information about 108 putative W-linked contigs (Genbank accession number GBAE01000000) from *G*. *affinis*, one positive control, and three sequences from *Oryzias hubbsi*. Likelihood of differential expression (DE) calculated in Bayseq v1.6.0, length of contigs in bp and absolute read count for each sequenced pool (F1-M6, F = female and M = male).
(DOCX)

S3 Table ENSEMBL results of Gaf88 BLAT search against platyfish genome (*Xiphophorus maculatus*) sorted by E-value. (DOCX)

S1 Fig S1a: Primer design for species confirmation. Multalin (v 5.4.1; [42]) alignment 5´- 3´ of the COI gene of *G*. *affinis* (Gaf: JN026704.1) and *G*. *holbrooki* (Gho: JN026706.1). The primers are marked in bold and underlined, sequence differences in red. Primers were chosen to give maximum melting temperature differences between both species (Gaf: 66.0/68.0°C, Gho: 60.4/67.4°C). Alignment parameters: Symbol comparison table: blosum62, Gap weight: 12, Gap length weight: 2. **S1b: Species-specific amplification COI primers**. PCR amplification of 327bp of the COI gene in *G*. *affinis* and *G*. *holbrooki* with Gaf primers (Gaf_F 66.0°C, Gaf_R 68.0°C) with a temperature gradient from 46–66°C. Due to the huge differences in T_m_ of the chosen primer sequences between both species (Gho: 60.4/67.4°C), there is hardly any product visible in *G*. *holbrooki* from 60°C upwards. 1.5% agarose gel, 0.5%TBE, 5V/cm.
(DOCX)

S2 Fig NGS coverage of Gaf88. The number of female reads mapping to contig23199X (Gaf88). The window scale is 0–100 reads and the length of contig in base pairs is shown by the top scale bar. The blue lines indicate primer locations. Male coverage is 0 (not shown).
(DOCX)

S3 Fig Alignment of the aminomethyl-transferase (*amt*) gene of *G*. *affinis* with *X*. *maculatus*. Multalin (v 5.4.1; [42]) alignment 5´- 3´ of *Gambusia affinis* consensus sequence with aminomethyl-transferase (*amt*) gene of *Xiphophorus maculatus* (ENSXMAT00000019396) showing a query coverage of 93% and a sequence identity of 90%. The sequencing primers are marked in bold and underlined, sequence differences in red. Lilac = untranscribed regions (UTR), black = introns, blue = exons, light yellow indicates the sequence of contig23199X (Gaf88) from the transcriptome of *G*. *affinis* in the 3´UTR region of the *X*. *maculatus amt gene*. Alignment parameters: Symbol comparison table: blosum62, Gap weight: 12, Gap length weight: 2.
(DOCX)
