# Supplementary material for: A Transcriptome Derived Female-Specific Marker from the Invasive Western Mosquitofish (Gambusia affinis)
Source: PLoS One. 2015 Feb 23;10(2):e0118214. doi: 10.1371/journal.pone.0118214 (PMC4338254; doi:10.1371/journal.pone.0118214)
Supplement: S1 Table — (DOCX) [file pone.0118214.s001.docx]

Table S1: NCBI BLAST of amplified COI sequences for species confirmation.

| **Seq name** | **Accession** | **Description** | [**Max score**](http://blast.ncbi.nlm.nih.gov/Blast.cgi?CMD=Get&ALIGNMENTS=100&ALIGNMENT_VIEW=Pairwise&BLAST_SPEC=WGS&DATABASE_SORT=0&DESCRIPTIONS=100&FIRST_QUERY_NUM=0&FORMAT_OBJECT=Alignment&FORMAT_PAGE_TARGET=&FORMAT_TYPE=HTML&GET_SEQUENCE=yes&I_THRESH=&MASK_CHAR=2&MASK_COLOR=1&NEW_VIEW=yes&NUM_OVERVIEW=100&OLD_BLAST=false&PAGE=Nucleotides&QUERY_INDEX=0&QUERY_NUMBER=0&RESULTS_PAGE_TARGET=&RID=WGY2D04P01S&SHOW_LINKOUT=yes&SHOW_OVERVIEW=yes&STEP_NUMBER=&WORD_SIZE=11&WWW_BLAST_TYPE_URL=&DISPLAY_SORT=1&HSP_SORT=1) | [**Total score**](http://blast.ncbi.nlm.nih.gov/Blast.cgi?CMD=Get&ALIGNMENTS=100&ALIGNMENT_VIEW=Pairwise&BLAST_SPEC=WGS&DATABASE_SORT=0&DESCRIPTIONS=100&FIRST_QUERY_NUM=0&FORMAT_OBJECT=Alignment&FORMAT_PAGE_TARGET=&FORMAT_TYPE=HTML&GET_SEQUENCE=yes&I_THRESH=&MASK_CHAR=2&MASK_COLOR=1&NEW_VIEW=yes&NUM_OVERVIEW=100&OLD_BLAST=false&PAGE=Nucleotides&QUERY_INDEX=0&QUERY_NUMBER=0&RESULTS_PAGE_TARGET=&RID=WGY2D04P01S&SHOW_LINKOUT=yes&SHOW_OVERVIEW=yes&STEP_NUMBER=&WORD_SIZE=11&WWW_BLAST_TYPE_URL=&DISPLAY_SORT=2&HSP_SORT=1) | [**Query coverage**](http://blast.ncbi.nlm.nih.gov/Blast.cgi?CMD=Get&ALIGNMENTS=100&ALIGNMENT_VIEW=Pairwise&BLAST_SPEC=WGS&DATABASE_SORT=0&DESCRIPTIONS=100&FIRST_QUERY_NUM=0&FORMAT_OBJECT=Alignment&FORMAT_PAGE_TARGET=&FORMAT_TYPE=HTML&GET_SEQUENCE=yes&I_THRESH=&MASK_CHAR=2&MASK_COLOR=1&NEW_VIEW=yes&NUM_OVERVIEW=100&OLD_BLAST=false&PAGE=Nucleotides&QUERY_INDEX=0&QUERY_NUMBER=0&RESULTS_PAGE_TARGET=&RID=WGY2D04P01S&SHOW_LINKOUT=yes&SHOW_OVERVIEW=yes&STEP_NUMBER=&WORD_SIZE=11&WWW_BLAST_TYPE_URL=&DISPLAY_SORT=4&HSP_SORT=0) | [**E value**](http://blast.ncbi.nlm.nih.gov/Blast.cgi?CMD=Get&ALIGNMENTS=100&ALIGNMENT_VIEW=Pairwise&BLAST_SPEC=WGS&DATABASE_SORT=0&DESCRIPTIONS=100&FIRST_QUERY_NUM=0&FORMAT_OBJECT=Alignment&FORMAT_PAGE_TARGET=&FORMAT_TYPE=HTML&GET_SEQUENCE=yes&I_THRESH=&MASK_CHAR=2&MASK_COLOR=1&NEW_VIEW=yes&NUM_OVERVIEW=100&OLD_BLAST=false&PAGE=Nucleotides&QUERY_INDEX=0&QUERY_NUMBER=0&RESULTS_PAGE_TARGET=&RID=WGY2D04P01S&SHOW_LINKOUT=yes&SHOW_OVERVIEW=yes&STEP_NUMBER=&WORD_SIZE=11&WWW_BLAST_TYPE_URL=&DISPLAY_SORT=0&HSP_SORT=0) | [**Max ident**](http://blast.ncbi.nlm.nih.gov/Blast.cgi?CMD=Get&ALIGNMENTS=100&ALIGNMENT_VIEW=Pairwise&BLAST_SPEC=WGS&DATABASE_SORT=0&DESCRIPTIONS=100&FIRST_QUERY_NUM=0&FORMAT_OBJECT=Alignment&FORMAT_PAGE_TARGET=&FORMAT_TYPE=HTML&GET_SEQUENCE=yes&I_THRESH=&MASK_CHAR=2&MASK_COLOR=1&NEW_VIEW=yes&NUM_OVERVIEW=100&OLD_BLAST=false&PAGE=Nucleotides&QUERY_INDEX=0&QUERY_NUMBER=0&RESULTS_PAGE_TARGET=&RID=WGY2D04P01S&SHOW_LINKOUT=yes&SHOW_OVERVIEW=yes&STEP_NUMBER=&WORD_SIZE=11&WWW_BLAST_TYPE_URL=&DISPLAY_SORT=3&HSP_SORT=3) |
| --- | --- | --- | --- | --- | --- | --- | --- |
| Gaf_f21 | [JN026704.1](http://www.ncbi.nlm.nih.gov/nucleotide/339769711?report=genbank&log$=nucltop&blast_rank=1&RID=WH26V3A901S) | *Gambusia affinis* voucher NAFF 4462 cytochrome oxidase subunit 1 (COI) gene, partial cds; mitochondrial | [551](http://blast.ncbi.nlm.nih.gov/Blast.cgi) | 551 | 99% | 1e-153 | 100% |
| Gho_f20 | [JN026708.1](http://www.ncbi.nlm.nih.gov/nucleotide/339769719?report=genbank&log$=nucltop&blast_rank=1&RID=WH2H2RY801S) | *Gambusia holbrooki* voucher NAFF 3027 cytochrome oxidase subunit 1 (COI) gene, partial cds; mitochondrial | [502](http://blast.ncbi.nlm.nih.gov/Blast.cgi) | 502 | 100% | 5e-139 | 100% |
