# Supplementary material for: A Transcriptome Derived Female-Specific Marker from the Invasive Western Mosquitofish (Gambusia affinis)
Source: PLoS One. 2015 Feb 23;10(2):e0118214. doi: 10.1371/journal.pone.0118214 (PMC4338254; doi:10.1371/journal.pone.0118214)
Supplement: S2 Table — Likelihood of differential expression (DE) calculated in Bayseq v1.6.0, length of contigs in bp and absolute read count for each sequenced pool (F1-M6, F = female and M = male). (DOCX) [file pone.0118214.s002.docx]

Table S2: Information about 108 putative W-linked contigs (Genbank accession number GBAE01000000) from *G. affinis*, one positive control, and three sequences from *Oryzias hubbsi*. Likelihood of differential expression (DE) calculated in Bayseq v1.6.0, length of contigs in bp and absolute read count for each sequenced pool (F1-M6, F=female and M=male).

| **Name** | **DE** | **Length** | **F1** | **F2** | **F3** | **F4** | **F5** | **F6** | **M1** | **M2** | **M3** | **M4** | **M5** | **M6** | **comment** |
| --- | --- | --- | --- | --- | --- | --- | --- | --- | --- | --- | --- | --- | --- | --- | --- |
| contig1529X | 1.00 | 547 | 30 | 20 | 35 | 26 | 14 | 11 | 0 | 0 | 0 | 0 | 0 | 0 |  |
| contig2408X | 1.00 | 658 | 56 | 22 | 30 | 23 | 25 | 14 | 0 | 0 | 0 | 0 | 0 | 0 |  |
| contig3509X | 1.00 | 1258 | 259 | 151 | 282 | 313 | 299 | 307 | 0 | 0 | 0 | 0 | 0 | 0 |  |
| contig3528X | 0.99 | 2801 | 1236 | 829 | 1376 | 1261 | 1398 | 1135 | 425 | 380 | 521 | 362 | 433 | 500 | OLb06.11h |
| contig4585X | 1.00 | 788 | 37 | 25 | 36 | 22 | 32 | 27 | 0 | 0 | 0 | 0 | 0 | 0 | Bacterial |
| contig5753X | 1.00 | 511 | 133 | 104 | 124 | 60 | 106 | 75 | 0 | 0 | 0 | 0 | 0 | 0 | Bacterial |
| contig7264X | 1.00 | 505 | 418 | 470 | 712 | 577 | 686 | 632 | 0 | 0 | 0 | 0 | 0 | 0 |  |
| contig10051X | 1.00 | 772 | 23 | 20 | 14 | 20 | 13 | 12 | 0 | 0 | 0 | 0 | 0 | 0 |  |
| contig10609X | 1.00 | 544 | 17 | 15 | 9 | 13 | 8 | 8 | 0 | 0 | 0 | 0 | 0 | 0 |  |
| contig11246X | 1.00 | 2275 | 88 | 56 | 84 | 47 | 94 | 52 | 0 | 0 | 0 | 0 | 0 | 0 | Bacterial |
| contig11338X | 1.00 | 1843 | 88 | 58 | 108 | 55 | 46 | 29 | 0 | 0 | 0 | 0 | 0 | 0 |  |
| contig11351X | 1.00 | 633 | 174 | 100 | 249 | 217 | 294 | 142 | 0 | 0 | 0 | 0 | 0 | 0 |  |
| contig11798X | 1.00 | 672 | 0 | 26 | 16 | 10 | 12 | 13 | 0 | 0 | 0 | 0 | 0 | 0 |  |
| contig12210X | 1.00 | 1031 | 39 | 19 | 28 | 6 | 34 | 16 | 0 | 0 | 0 | 0 | 0 | 0 | Bacterial |
| contig12355X | 1.00 | 820 | 16 | 15 | 11 | 11 | 6 | 21 | 0 | 0 | 0 | 0 | 0 | 0 |  |
| contig12489X | 1.00 | 597 | 2591 | 1431 | 3153 | 2219 | 3359 | 1953 | 0 | 0 | 0 | 0 | 0 | 0 |  |
| contig12512X | 1.00 | 501 | 15 | 22 | 27 | 5 | 3 | 4 | 0 | 0 | 0 | 0 | 0 | 0 |  |
| contig12627X | 1.00 | 581 | 83 | 101 | 1 | 0 | 129 | 21 | 0 | 0 | 0 | 0 | 0 | 0 |  |
| contig12811X | 1.00 | 1322 | 144 | 161 | 158 | 97 | 165 | 166 | 0 | 0 | 0 | 0 | 0 | 0 |  |
| contig12825X | 1.00 | 944 | 7 | 20 | 79 | 28 | 41 | 12 | 0 | 0 | 0 | 0 | 0 | 0 |  |
| contig12980X | 1.00 | 621 | 39 | 32 | 47 | 46 | 73 | 39 | 0 | 0 | 0 | 0 | 0 | 0 |  |
| contig12981X | 1.00 | 692 | 37 | 28 | 41 | 34 | 43 | 28 | 0 | 0 | 0 | 0 | 0 | 0 |  |
| contig13072X | 1.00 | 537 | 49 | 33 | 48 | 14 | 50 | 28 | 0 | 0 | 0 | 0 | 0 | 0 | Bacterial |
| contig13318X | 1.00 | 523 | 16 | 9 | 21 | 16 | 14 | 14 | 0 | 0 | 0 | 0 | 0 | 0 |  |
| contig13424X | 1.00 | 717 | 8 | 5 | 19 | 18 | 13 | 36 | 0 | 0 | 0 | 0 | 0 | 0 |  |
| contig13439X | 1.00 | 524 | 40 | 21 | 31 | 54 | 34 | 40 | 0 | 0 | 0 | 0 | 0 | 0 |  |
| contig13489X | 1.00 | 729 | 10 | 10 | 34 | 33 | 22 | 22 | 0 | 0 | 0 | 0 | 0 | 0 |  |
| contig13556X | 1.00 | 955 | 51 | 58 | 118 | 78 | 98 | 60 | 0 | 0 | 0 | 0 | 0 | 0 |  |
| contig13676X | 1.00 | 785 | 15 | 5 | 17 | 12 | 22 | 37 | 0 | 0 | 0 | 0 | 0 | 0 |  |
| contig14343X | 1.00 | 624 | 20 | 32 | 28 | 5 | 16 | 12 | 0 | 0 | 0 | 0 | 0 | 0 | Bacterial |
| contig15716X | 0.02 | 993 | 1005 | 1435 | 766 | 541 | 1181 | 737 | 1062 | 387 | 329 | 184 | 368 | 407 | Pos. control |
| contig17284X | 1.00 | 3362 | 311 | 120 | 291 | 804 | 239 | 425 | 0 | 0 | 0 | 0 | 0 | 0 |  |
| contig17851X | 1.00 | 2457 | 131 | 116 | 107 | 168 | 188 | 138 | 0 | 0 | 0 | 0 | 0 | 0 |  |
| contig17854X | 1.00 | 2443 | 480 | 455 | 134 | 467 | 142 | 147 | 0 | 0 | 0 | 0 | 0 | 0 |  |
| contig18087X | 1.00 | 2244 | 98 | 45 | 97 | 98 | 133 | 92 | 0 | 0 | 0 | 0 | 0 | 0 |  |
| contig18892X | 1.00 | 1766 | 47 | 59 | 83 | 123 | 137 | 104 | 0 | 0 | 0 | 0 | 0 | 0 |  |
| contig18975X | 1.00 | 1721 | 125 | 96 | 105 | 89 | 67 | 57 | 0 | 0 | 0 | 0 | 0 | 0 |  |
| contig19032X | 1.00 | 1702 | 121 | 32 | 70 | 15 | 38 | 185 | 0 | 0 | 0 | 0 | 0 | 0 |  |
| contig19153X | 1.00 | 1657 | 91 | 76 | 109 | 158 | 185 | 49 | 0 | 0 | 0 | 0 | 0 | 0 |  |
| contig19279X | 1.00 | 1609 | 174 | 174 | 234 | 222 | 216 | 158 | 0 | 0 | 0 | 0 | 0 | 0 |  |
| contig19387X | 1.00 | 1566 | 235 | 147 | 299 | 269 | 372 | 228 | 0 | 0 | 0 | 0 | 0 | 0 |  |
| contig19488X | 1.00 | 1534 | 46 | 32 | 35 | 51 | 58 | 42 | 0 | 0 | 0 | 0 | 0 | 0 |  |
| contig19510X | 1.00 | 1525 | 151 | 139 | 212 | 163 | 205 | 124 | 0 | 0 | 0 | 0 | 0 | 0 |  |
| contig19588X | 1.00 | 1501 | 4042 | 2300 | 4537 | 5112 | 4824 | 4201 | 0 | 0 | 0 | 0 | 0 | 0 |  |
| contig19668X | 1.00 | 1475 | 52 | 55 | 2 | 2 | 37 | 1 | 0 | 0 | 0 | 0 | 0 | 0 |  |
| contig20285X | 1.00 | 1292 | 64 | 24 | 33 | 26 | 48 | 62 | 0 | 0 | 0 | 0 | 0 | 0 |  |
| contig20352X | 1.00 | 1273 | 85 | 109 | 209 | 80 | 122 | 94 | 0 | 0 | 0 | 0 | 0 | 0 |  |
| contig20353X | 1.00 | 1270 | 57 | 33 | 102 | 62 | 84 | 55 | 0 | 0 | 0 | 0 | 0 | 0 |  |
| contig20429X | 1.00 | 1251 | 105 | 82 | 41 | 15 | 21 | 88 | 0 | 0 | 0 | 0 | 0 | 0 |  |
| contig20561X | 1.00 | 1226 | 61 | 8 | 9 | 50 | 39 | 46 | 0 | 0 | 0 | 0 | 0 | 0 |  |
| contig20710X | 1.00 | 1196 | 552 | 408 | 418 | 621 | 582 | 339 | 0 | 0 | 0 | 0 | 0 | 0 |  |
| contig20738X | 1.00 | 1186 | 160 | 84 | 208 | 161 | 120 | 220 | 0 | 0 | 0 | 0 | 0 | 0 |  |
| contig20769X | 1.00 | 1183 | 24 | 22 | 54 | 28 | 47 | 41 | 0 | 0 | 0 | 0 | 0 | 0 | not tested |
| contig20853X | 1.00 | 1164 | 43 | 32 | 67 | 44 | 40 | 19 | 0 | 0 | 0 | 0 | 0 | 0 | not tested |
| contig20934X | 1.00 | 1150 | 83 | 51 | 73 | 20 | 36 | 34 | 0 | 0 | 0 | 0 | 0 | 0 | not tested |
| contig21193X | 1.00 | 1093 | 28 | 28 | 0 | 1 | 36 | 51 | 0 | 0 | 0 | 0 | 0 | 0 | not tested |
| contig21198X | 1.00 | 1098 | 42 | 14 | 5 | 59 | 46 | 27 | 0 | 0 | 0 | 0 | 0 | 0 | not tested |
| contig21295X | 1.00 | 1080 | 6 | 16 | 40 | 35 | 25 | 12 | 0 | 0 | 0 | 0 | 0 | 0 | not tested |
| contig21420X | 1.00 | 1055 | 25 | 37 | 67 | 63 | 62 | 58 | 0 | 0 | 0 | 0 | 0 | 0 | not tested |
| contig21441X | 1.00 | 1050 | 8 | 8 | 28 | 49 | 45 | 24 | 0 | 0 | 0 | 0 | 0 | 0 | not tested |
| contig21743X | 1.00 | 995 | 44 | 41 | 78 | 57 | 75 | 58 | 0 | 0 | 0 | 0 | 0 | 0 | not tested |
| contig21765X | 1.00 | 989 | 18 | 14 | 60 | 28 | 31 | 28 | 0 | 0 | 0 | 0 | 0 | 0 | not tested |
| contig21773X | 1.00 | 991 | 65 | 23 | 26 | 46 | 44 | 53 | 0 | 0 | 0 | 0 | 0 | 0 | not tested |
| contig21808X | 1.00 | 987 | 16 | 28 | 32 | 20 | 35 | 8 | 0 | 0 | 0 | 0 | 0 | 0 | not tested |
| contig21898X | 1.00 | 973 | 69 | 42 | 50 | 111 | 102 | 66 | 0 | 0 | 0 | 0 | 0 | 0 | not tested |
| contig21982X | 1.00 | 956 | 12 | 20 | 24 | 40 | 60 | 15 | 0 | 0 | 0 | 0 | 0 | 0 | not tested |
| contig21989X | 1.00 | 956 | 156 | 49 | 218 | 150 | 314 | 185 | 0 | 0 | 0 | 0 | 0 | 0 | not tested |
| contig22071X | 1.00 | 942 | 25 | 1 | 22 | 11 | 32 | 11 | 0 | 0 | 0 | 0 | 0 | 0 | not tested |
| contig22131X | 1.00 | 932 | 104 | 78 | 88 | 114 | 149 | 102 | 0 | 0 | 0 | 0 | 0 | 0 | not tested |
| contig22307X | 1.00 | 904 | 19 | 12 | 36 | 29 | 27 | 23 | 0 | 0 | 0 | 0 | 0 | 0 | not tested |
| contig22596X | 1.00 | 861 | 8 | 0 | 23 | 26 | 2 | 26 | 0 | 0 | 0 | 0 | 0 | 0 | not tested |
| contig22730X | 1.00 | 839 | 110 | 75 | 33 | 24 | 81 | 32 | 0 | 0 | 0 | 0 | 0 | 0 | not tested |
| contig22786X | 1.00 | 834 | 39 | 21 | 21 | 59 | 47 | 31 | 0 | 0 | 0 | 0 | 0 | 0 | not tested |
| contig22853X | 1.00 | 822 | 23 | 21 | 16 | 25 | 29 | 21 | 0 | 0 | 0 | 0 | 0 | 0 | not tested |
| contig23031X | 1.00 | 802 | 39 | 19 | 35 | 9 | 31 | 28 | 0 | 0 | 0 | 0 | 0 | 0 | Bacterial |
| contig23045X | 1.00 | 800 | 44 | 16 | 72 | 109 | 108 | 73 | 0 | 0 | 0 | 0 | 0 | 0 |  |
| contig23134X | 1.00 | 788 | 17 | 33 | 33 | 35 | 36 | 35 | 0 | 0 | 0 | 0 | 0 | 0 |  |
| contig23199X | 1.00 | 780 | 39 | 37 | 31 | 15 | 34 | 15 | 0 | 0 | 0 | 0 | 0 | 0 | Gaf88 |
| contig23206X | 1.00 | 778 | 20 | 16 | 35 | 27 | 36 | 33 | 0 | 0 | 0 | 0 | 0 | 0 |  |
| contig23371X | 1.00 | 756 | 141 | 141 | 133 | 50 | 123 | 93 | 0 | 0 | 0 | 0 | 0 | 0 | Bacterial |
| contig23779X | 1.00 | 708 | 12 | 19 | 35 | 13 | 16 | 16 | 0 | 0 | 0 | 0 | 0 | 0 |  |
| contig24035X | 1.00 | 679 | 587 | 506 | 915 | 691 | 1010 | 652 | 0 | 0 | 0 | 0 | 0 | 0 |  |
| contig24053X | 1.00 | 675 | 29 | 9 | 38 | 42 | 26 | 34 | 0 | 0 | 0 | 0 | 0 | 0 |  |
| contig24094X | 1.00 | 671 | 14 | 14 | 27 | 23 | 28 | 18 | 0 | 0 | 0 | 0 | 0 | 0 |  |
| contig24113X | 1.00 | 666 | 19 | 12 | 20 | 30 | 32 | 65 | 0 | 0 | 0 | 0 | 0 | 0 |  |
| contig24124X | 1.00 | 667 | 40 | 47 | 53 | 81 | 133 | 40 | 0 | 0 | 0 | 0 | 0 | 0 |  |
| contig24282X | 1.00 | 648 | 15 | 14 | 20 | 19 | 49 | 18 | 0 | 0 | 0 | 0 | 0 | 0 |  |
| contig24333X | 1.00 | 642 | 127 | 53 | 117 | 86 | 125 | 95 | 0 | 0 | 0 | 0 | 0 | 0 |  |
| contig24348X | 1.00 | 640 | 241 | 123 | 380 | 206 | 497 | 246 | 0 | 0 | 0 | 0 | 0 | 0 |  |
| contig24446X | 1.00 | 630 | 44 | 13 | 33 | 30 | 35 | 24 | 0 | 0 | 0 | 0 | 0 | 0 |  |
| contig24519X | 1.00 | 621 | 2102 | 921 | 1861 | 2506 | 2160 | 2103 | 0 | 0 | 0 | 0 | 0 | 0 |  |
| contig24521X | 1.00 | 622 | 56 | 35 | 48 | 67 | 77 | 41 | 0 | 0 | 0 | 0 | 0 | 0 |  |
| contig24559X | 1.00 | 617 | 12 | 19 | 39 | 25 | 41 | 16 | 0 | 0 | 0 | 0 | 0 | 0 |  |
| contig24584X | 1.00 | 614 | 14 | 11 | 18 | 27 | 32 | 20 | 0 | 0 | 0 | 0 | 0 | 0 |  |
| contig24678X | 1.00 | 603 | 35 | 50 | 73 | 72 | 114 | 66 | 0 | 0 | 0 | 0 | 0 | 0 |  |
| contig24683X | 1.00 | 602 | 122 | 52 | 116 | 100 | 54 | 91 | 0 | 0 | 0 | 0 | 0 | 0 |  |
| contig24707X | 1.00 | 602 | 25 | 39 | 27 | 28 | 52 | 27 | 0 | 0 | 0 | 0 | 0 | 0 |  |
| contig24712X | 1.00 | 600 | 26 | 20 | 11 | 30 | 22 | 35 | 0 | 0 | 0 | 0 | 0 | 0 |  |
| contig24845X | 1.00 | 586 | 16 | 17 | 22 | 24 | 20 | 9 | 0 | 0 | 0 | 0 | 0 | 0 |  |
| contig24873X | 1.00 | 582 | 19 | 13 | 20 | 18 | 35 | 26 | 0 | 0 | 0 | 0 | 0 | 0 |  |
| contig24882X | 1.00 | 577 | 88 | 89 | 166 | 86 | 97 | 60 | 0 | 0 | 0 | 0 | 0 | 0 |  |
| contig24947X | 1.00 | 575 | 51 | 39 | 119 | 81 | 101 | 78 | 0 | 0 | 0 | 0 | 0 | 0 |  |
| contig25025X | 1.00 | 566 | 58 | 51 | 60 | 28 | 46 | 53 | 0 | 0 | 0 | 0 | 0 | 0 |  |
| contig25055X | 1.00 | 564 | 5 | 21 | 6 | 14 | 7 | 10 | 0 | 0 | 0 | 0 | 0 | 0 |  |
| contig25193X | 1.00 | 549 | 17 | 17 | 21 | 13 | 10 | 17 | 0 | 0 | 0 | 0 | 0 | 0 |  |
| contig25242X | 1.00 | 542 | 31 | 24 | 40 | 30 | 48 | 28 | 0 | 0 | 0 | 0 | 0 | 0 |  |
| contig25293X | 1.00 | 524 | 11 | 2 | 12 | 22 | 17 | 20 | 0 | 0 | 0 | 0 | 0 | 0 |  |
| contig25353X | 1.00 | 526 | 15 | 2 | 41 | 4 | 28 | 9 | 0 | 0 | 0 | 0 | 0 | 0 |  |
| contig25571X | 1.00 | 503 | 18 | 9 | 7 | 13 | 23 | 11 | 0 | 0 | 0 | 0 | 0 | 0 |  |
| contig25645X | 1.00 | 500 | 74 | 86 | 88 | 35 | 56 | 60 | 0 | 0 | 0 | 0 | 0 | 0 |  |
| contig34498X | 0.98 | 609 | 864 | 598 | 1052 | 926 | 1157 | 820 | 415 | 247 | 314 | 248 | 259 | 410 | br8179 |
| contig38307X | 1.00 | 348 | 6545 | 6291 | 6058 | 3742 | 5160 | 4281 | 1282 | 1164 | 1038 | 834 | 1173 | 2022 | OLb22.11h |
