# Supplementary material for: A Transcriptome Derived Female-Specific Marker from the Invasive Western Mosquitofish (Gambusia affinis)
Source: PLoS One. 2015 Feb 23;10(2):e0118214. doi: 10.1371/journal.pone.0118214 (PMC4338254; doi:10.1371/journal.pone.0118214)
Supplement: S3 Table — (DOCX) [file pone.0118214.s003.docx]

Table S3: ENSEMBL results of Gaf88 BLAT search against platyfish genome (*Xiphophorus maculatus*) sorted by E-value.

| **Query start** | **Query end** | **Scaffold name** | **Start** | **End** | **Ori** | **Score** | **E-val** | **%ID** | **Length** |
| --- | --- | --- | --- | --- | --- | --- | --- | --- | --- |
|  |  |  |  |  |  |  |  |  |  |
| 444 | 779 | JH556705.1 | 1.171.940 | 1.172.279 | + | 1.406 | 3.4e-241 | 91.23 | 342 |
| 55 | 175 | JH556705.1 | 1.172.617 | 1.172.737 | + | 544 | 4.9e-88 | 94.21 | 121 |
| 198 | 318 | JH556705.1 | 1.172.455 | 1.172.580 | + | 525 | 1.2e-84 | 91.27 | 126 |
| 332 | 443 | JH556705.1 | 1.172.312 | 1.172.423 | + | 508 | 1.3e-81 | 95.54 | 112 |
