# Supplementary material for: A Transcriptome Derived Female-Specific Marker from the Invasive Western Mosquitofish (Gambusia affinis)
Source: PLoS One. 2015 Feb 23;10(2):e0118214. doi: 10.1371/journal.pone.0118214 (PMC4338254; doi:10.1371/journal.pone.0118214)
Supplement: S1 Fig — Multalin (v 5.4.1; [42]) alignment 5´- 3´ of the COI gene of G. affinis (Gaf: JN026704.1) and G. holbrooki (Gho: JN026706.1). The primers are marked in bold and underlined, sequence differences in red. Primers were chosen to give maximum melting temperature differences between both species (Gaf: 66.0/68.0°C, Gho: 60.4/67.4°C). Alignment parameters: Symbol comparison table: blosum62, Gap weight: 12, Gap length weight: 2. S1b: Species-specific amplification COI primers. PCR amplification of 327bp of the COI gene in G. affinis and G. holbrooki with Gaf primers (Gaf_F 66.0°C, Gaf_R 68.0°C) with a temperature gradient from 46–66°C. Due to the huge differences in Tm of the chosen primer sequences between both species (Gho: 60.4/67.4°C), there is hardly any product visible in G. holbrooki from 60°C upwards. 1.5% agarose gel, 0.5%TBE, 5V/cm. (DOCX) [file pone.0118214.s004.docx]

**
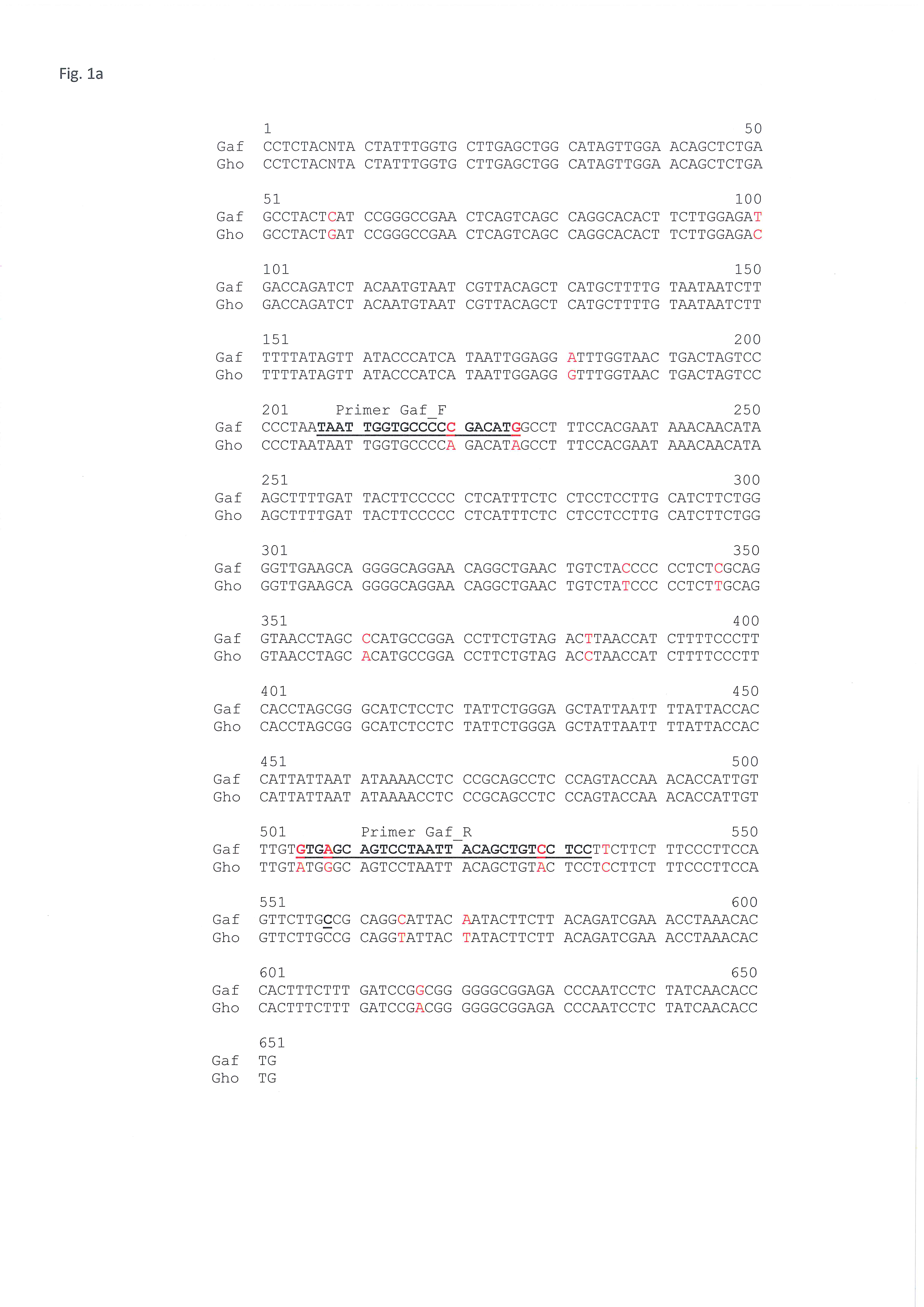
**

**Fig. S1a: Primer design for species confirmation.**

Multalin (v 5.4.1; [42]) alignment 5´- 3´ of the COI gene of *G. affinis* (Gaf: JN026704.1) and *G. holbrooki* (Gho: JN026706.1). The primers are marked in bold and underlined, sequence differences in red. Primers were chosen to give maximum melting temperature differences between both species (Gaf: 66.0/68.0 °C, Gho: 60.4/67.4 °C). Alignment parameters: Symbol comparison table: blosum62, Gap weight: 12, Gap length weight: 2.

**
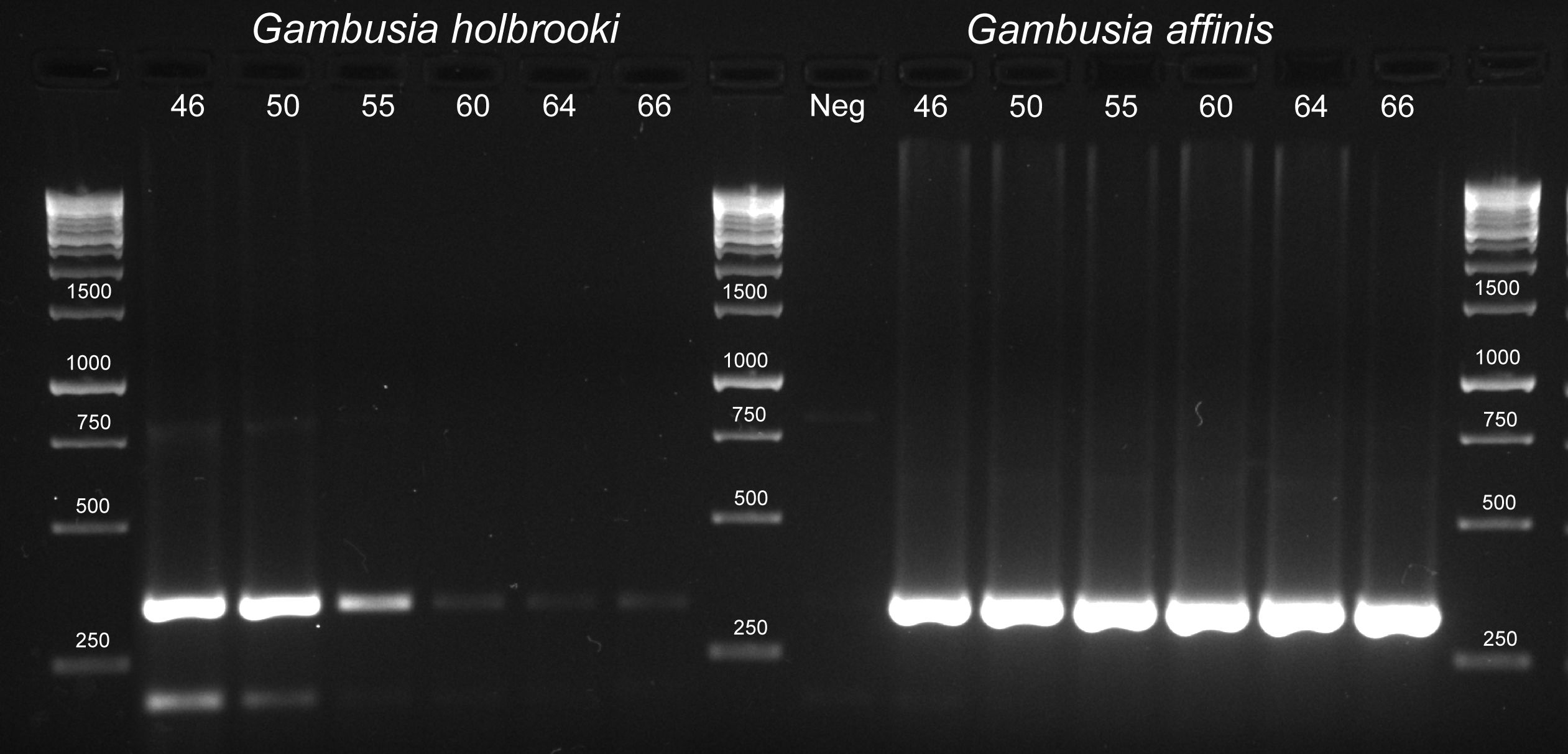
**

**Fig. S1b:** **Species-specific amplification COI primers.**

PCR amplification of 327bp of the COI gene in *G. affinis* and *G. holbrooki* with Gaf primers (Gaf_F 66.0 °C, Gaf_R 68.0 °C) with a temperature gradient from 46-66 °C. Due to the huge differences in T_m_ of the chosen primer sequences between both species (Gho: 60.4/67.4 °C), there is hardly any product visible in *G. holbrooki* from 60 °C upwards. 1.5% agarose gel, 0.5%TBE, 5V/cm.
