# Supplementary material for: A Transcriptome Derived Female-Specific Marker from the Invasive Western Mosquitofish (Gambusia affinis)
Source: PLoS One. 2015 Feb 23;10(2):e0118214. doi: 10.1371/journal.pone.0118214 (PMC4338254; doi:10.1371/journal.pone.0118214)
Supplement: S2 Fig — The number of female reads mapping to contig23199X (Gaf88). The window scale is 0–100 reads and the length of contig in base pairs is shown by the top scale bar. The blue lines indicate primer locations. Male coverage is 0 (not shown). (DOCX) [file pone.0118214.s005.docx]

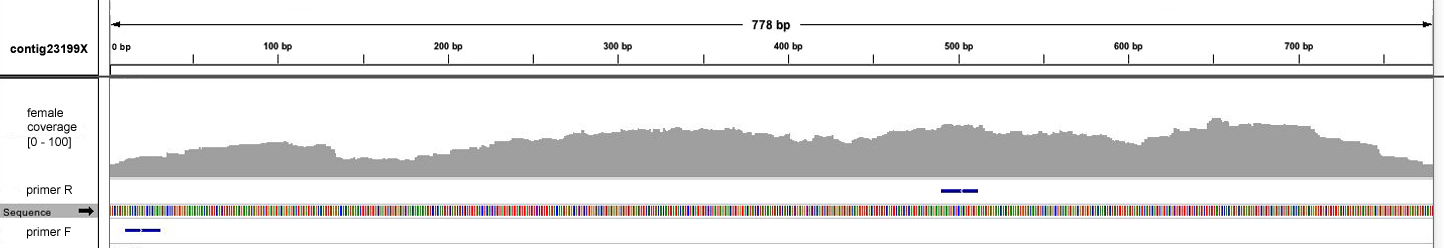


**Fig. S2:** **NGS coverage of Gaf88.**

The number of female reads mapping to contig23199X (Gaf88). The window scale is 0-100 reads and the length of contig in base pairs is shown by the top scale bar. The blue lines indicate primer locations. Male coverage is 0 (not shown).
