# Supplementary material for: A Transcriptome Derived Female-Specific Marker from the Invasive Western Mosquitofish (Gambusia affinis)
Source: PLoS One. 2015 Feb 23;10(2):e0118214. doi: 10.1371/journal.pone.0118214 (PMC4338254; doi:10.1371/journal.pone.0118214)
Supplement: S3 Fig — Multalin (v 5.4.1; [42]) alignment 5´- 3´ of Gambusia affinis consensus sequence with aminomethyl-transferase (amt) gene of Xiphophorus maculatus (ENSXMAT00000019396) showing a query coverage of 93% and a sequence identity of 90%. The sequencing primers are marked in bold and underlined, sequence differences in red. Lilac = untranscribed regions (UTR), black = introns, blue = exons, light yellow indicates the sequence of contig23199X (Gaf88) from the transcriptome of G. affinis in the 3´UTR region of the X. maculatus amt gene. Alignment parameters: Symbol comparison table: blosum62, Gap weight: 12, Gap length weight: 2. (DOCX) [file pone.0118214.s006.docx]

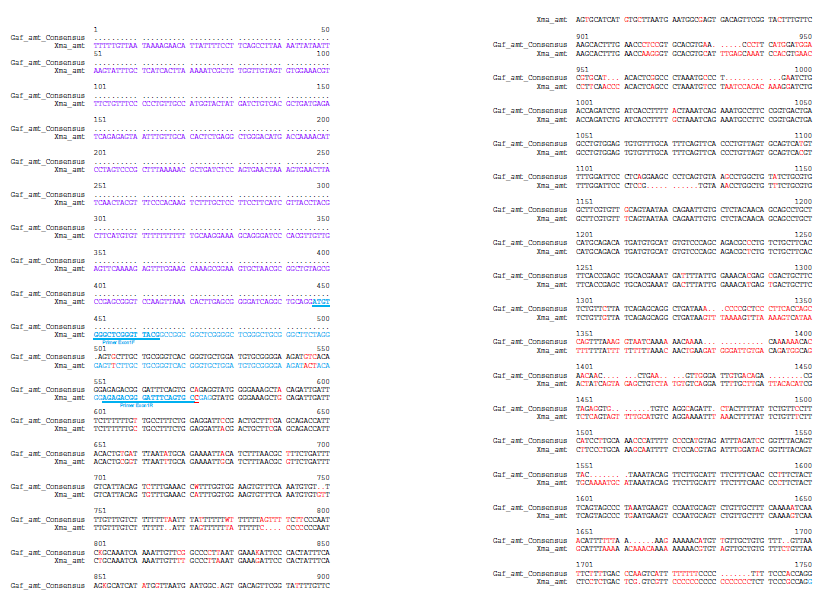


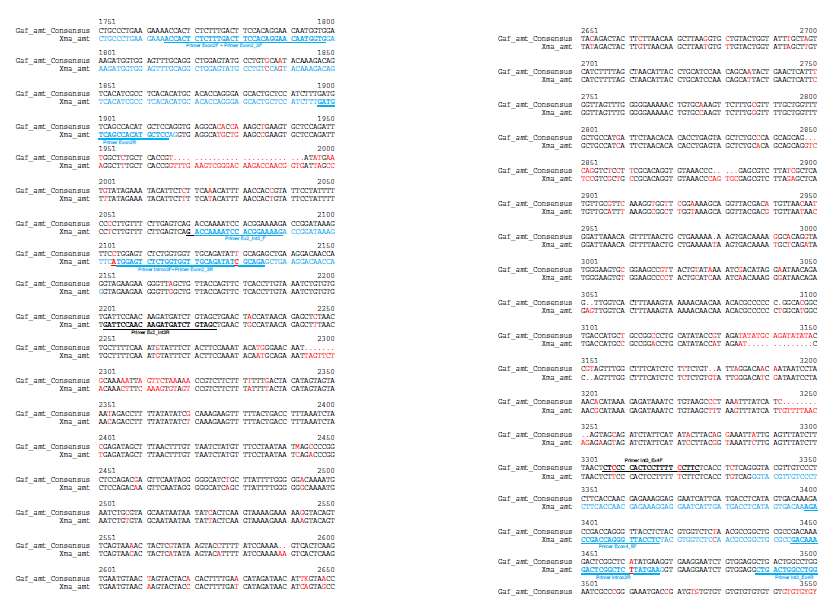


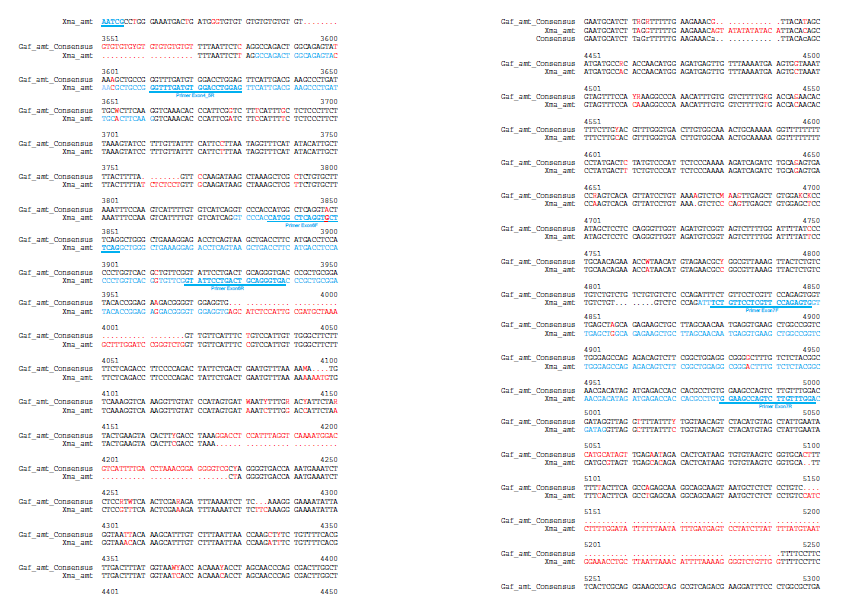


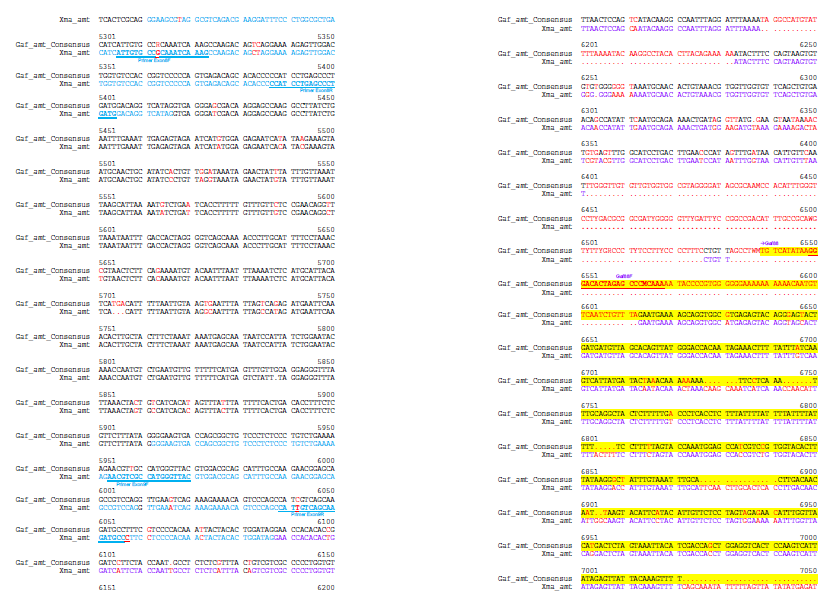


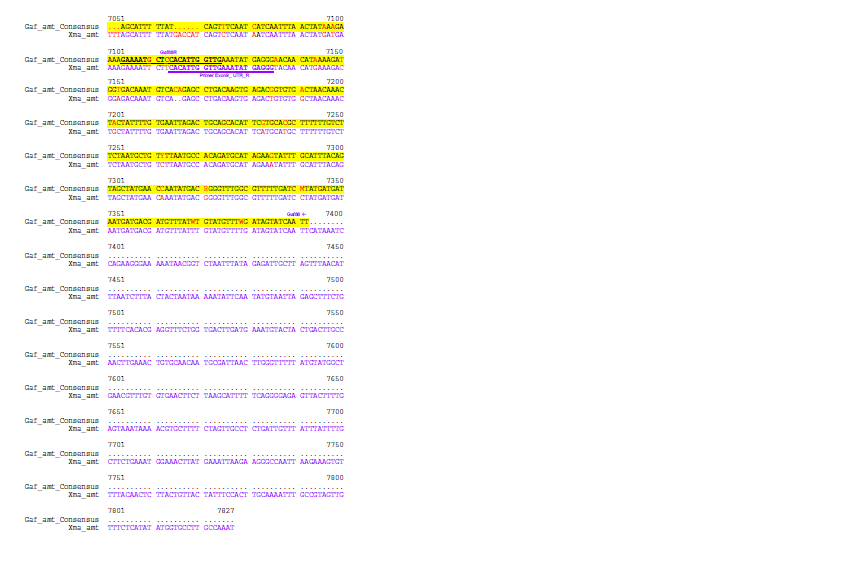


**Fig.S3: Alignment of the aminomethyl-transferase (*amt*) gene of *G. affinis* with *X. maculatus.***

Multalin (v 5.4.1; [42]) alignment 5´- 3´ of *Gambusia affinis* consensus sequence with aminomethyl-transferase (*amt*) gene of *Xiphophorus maculatus* (ENSXMAT00000019396) showing a query coverage of 93% and a sequence identity of 90%. The sequencing primers are marked in bold and underlined, sequence differences in red. Lilac = untranscribed regions (UTR), black = introns, blue = exons, light yellow indicates the sequence of contig23199X (Gaf88) from the transcriptome of *G. affinis* in the 3´UTR region of the *X. maculatus amt* gene. Alignment parameters: Symbol comparison table: blosum62, Gap weight: 12, Gap length weight: 2.
